# Supplementary material for: Local field potential sharp waves with diversified impact on cortical neuronal encoding of haptic input
Source: Sci Rep. 2024 Jul 2;14:15243. doi: 10.1038/s41598-024-65200-3 (PMC11219916; doi:10.1038/s41598-024-65200-3)
Supplement: Supplementary file 1 — Supplementary Information. [file 41598_2024_65200_MOESM1_ESM.pdf]

Supplementary Material

Title: Local field potential sharp waves with diversified impact on cortical neuronal encoding of haptic input

Authors: Sofie Skårup Kristensen & Henrik Jörntell

A) Mann-Whitney U test results (N=48): Comparing the distribution of evoked spikes when stimulation was preceded by an LFP-SPW (group A) with the distribution of evoked spikes when stimulation was not preceded by an LFP-SPW (group B) + effect size of neurons with alpha < 0.05.

Depressed (N=19):

1. Neuron#1: N(A) = 159 and N(B) = 161; U = 16976.0; P = 0.0; one-tailed; r=0.372
2. Neuron#2: N(A) = 82 and N(B) = 238; U = 11208.5; P = 0.004; one-tailed; r=0.154
3. Neuron#3: N(A) = 110 and N(B) = 450; U = 29211.5; P = 0.001; one-tailed; r=0.190
4. Neuron#4: N(A) = 113 and N(B) = 447; U = 28071.5; P = 0.007; one-tailed; r=0.105
5. Neuron#5: N(A) = 96 and N(B) = 464; U = 24452.5; P = 0.023; one-tailed; r=0.1787
6. Neuron#6: N(A) = 73 and N(B) = 487; U = 20086.5; P = 0.019; one-tailed; r=0.1875
7. Neuron#7: N(A) = 123 and N(B) = 439; U = 29967.0; P = 0.011; one-tailed; r=0.105
8. Neuron#8: N(A) = 78 and N(B) = 362; U = 17607.0; P = 0.0; one-tailed; r=0.189
9. Neuron#9: N(A) = 160 and N(B) = 620; U = 56350.5; P = 0.0; one-tailed; r=0.117
10. Neuron#10: N(A) = 171 and N(B) = 219; U = 21978.5; P = 0.001; one-tailed; r=0.136
11. Neuron#11: N(A) = 156 and N(B) = 234; U = 22110.0; P = 0.0; one-tailed; r=0.193
12. Neuron#12: N(A) = 146 and N(B) = 244; U = 20577.5; P = 0.002; one-tailed; r=0.134
13. Neuron#13: N(A) = 116 and N(B) = 274; U = 17909.0; P = 0.014; one-tailed; r=0.116
14. Neuron#14: N(A) = 169 and N(B) = 221; U = 24397.5; P = 0.0; one-tailed; r=0.298
15. Neuron#15: N(A) = 154 and N(B) = 236; U = 22605.0; P = 0.0; one-tailed; r=0.224
16. Neuron#16: N(A) = 138 and N(B) = 252; U = 20795.0; P = 0.0; one-tailed; r=0.173
17. Neuron#17: N(A) = 109 and N(B) = 281; U = 16786.0; P = 0.051; one-tailed; r=0.173
18. Neuron#18: N(A) = 122 and N(B) = 268; U = 14377.5; P = 0.016; one-tailed; r=0.108
19. Neuron#19: N(A) = 86 and N(B) = 123; U = 6439.0; P = 0.0; one-tailed; r=0.257

Excited (N=9):

1. Neuron#20: N(A) = 177 and N(B) = 383; U = 30099.0; P = 0.005; one-tailed; r=0.110
2. Neuron#21: N(A) = 17 and N(B) = 123; U = 747.5; P = 0.007; one-tailed; r=0.208
3. Neuron#22: N(A) = 29 and N(B) = 191; U = 2415.0; P = 0.033; one-tailed; r=0.117
4. Neuron#23: N(A) = 98 and N(B) = 292; U = 12244.5; P = 0.006; one-tailed; r=0.128
5. Neuron#24: N(A) = 28 and N(B) = 72; U = 808.0; P = 0.01; one-tailed; r=0.235
6. Neuron#25: N(A) = 76 and N(B) = 314; U = 10079.0; P = 0.005; one-tailed; r=0.128
7. Neuron#26: N(A) = 153 and N(B) = 237; U = 15353.0; P = 0.001; one-tailed; r=0.160
8. Neuron#27: N(A) = 152 and N(B) = 238; U = 16481.0; P = 0.055; one-tailed; r = 0.16
9. Neuron#28: N(A) = 122 and N(B) = 268; U = 14377.5; P = 0.016; one-tailed; r=0.108

No Effect (N=20):

1. Neuron#29: N(A) = 49 and N(B) = 271; U = 7059.5; P = 0.193; one-tailed
2. Neuron#30: N(A) = 134 and N(B) = 426; U = 30959.5; P = 0.061; one-tailed
3. Neuron#31: N(A) = 42 and N(B) = 518; U = 11860.0; P = 0.126; one-tailed
4. Neuron#32: N(A) = 62 and N(B) = 378; U = 12231.0; P = 0.219; one-tailed
5. Neuron#33: N(A) = 52 and N(B) = 338; U = 8258.0; P = 0.155; one-tailed
6. Neuron#34: N(A) = 189 and N(B) = 379; U = 35441.5; P = 0.606; one-tailed
7. Neuron#35: N(A) = 63 and N(B) = 116; U = 4050.5; P = 0.086; one-tailed
8. Neuron#36: N(A) = 156 and N(B) = 234; U = 19551.5; P = 0.063; one-tailed
9. Neuron#37: N(A) = 36 and N(B) = 184; U = 3572.0; P = 0.148; one-tailed
10. Neuron#38: N(A) = 63 and N(B) = 157; U = 5378.0; P = 0.067; one-tailed
11. Neuron#39: N(A) = 103 and N(B) = 287; U = 13978.0; P = 0.172; one-tailed
12. Neuron#40: N(A) = 79 and N(B) = 171; U = 6772.5; P = 0.526; one-tailed
13. Neuron#41: N(A) = 81 and N(B) = 309; U = 11740.5; P = 0.151; one-tailed
14. Neuron#42: N(A) = 58 and N(B) = 162; U = 4945.5; P = 0.243; one-tailed
15. Neuron#43: N(A) = 123 and N(B) = 267; U = 15260.0; P = 0.112; one-tailed
16. Neuron#44: N(A) = 101 and N(B) = 289; U = 15041.5; P = 0.315; one-tailed
17. Neuron#45: N(A) = 124 and N(B) = 266; U = 14864.5; P = 0.041; one-tailed
18. Neuron#46: N(A) = 38 and N(B) = 122; U = 2218.5; P = 0.276; one-tailed
19. Neuron#47: N(A) = 162 and N(B) = 228; U = 17763.5; P = 0.183; one-tailed
20. Neuron#48: N(A) = 108 and N(B) = 197; U = 10863.0; P = 0.324; one-tailed

B) Mann-Whitney U test results (N=38): Comparing the distribution of evoked spikes when stimulation was preceded by an LFP-SPW with coinciding ECoG-SPW (group A) with the distribution of evoked spikes when stimulation was preceded by an LFP-SPW without a coinciding ECoG-SPW (group B) + effect size of neurons with alpha < 0.05.

Depressed (N=4):

1. Neuron#30: N(A) = 47 and N(B) = 87; U = 2386.5; P = 0.047; one-tailed; r=0.141
2. Neuron#20: N(A) = 55 and N(B) = 122; U = 3970.0; P = 0.012; one-tailed; r=0.169
3. Neuron#27: N(A) = 34 and N(B) = 118; U = 2404.0; P = 0.031; one-tailed; r=0.131
4. Neuron#19: N(A) = 36 and N(B) = 50; U = 1001.0; P = 0.04; one-tailed; r=0.189

Excited (N=5):

1. Neuron#34: N(A) = 73 and N(B) = 116; U = 3422.0; P = 0.002; one-tailed; r=0.212
2. Neuron#43: N(A) = 17 and N(B) = 106; U = 467.0; P = 0.0; one-tailed; r=0.286
3. Neuron#44: N(A) = 23 and N(B) = 78; U = 681.5; P = 0.035; one-tailed; r=0.187
4. Neuron#45: N(A) = 30 and N(B) = 94; U = 1055.5; P = 0.013; one-tailed; r=0.213
5. Neuron#18: N(A) = 68 and N(B) = 54; U = 1458.5; P = 0.017; one-tailed; r=0.192

No Effect (N=29):

1. Neuron#29: N(A) = 15 and N(B) = 34; U = 208.5; P = 0.097; one-tailed
2. Neuron#1: N(A) = 33 and N(B) = 126; U = 2030.5; P = 0.364; one-tailed

3. Neuron#2: N(A) = 41 and N(B) = 41; U = 863.5; P = 0.362; one-tailed
4. Neuron#3: N(A) = 52 and N(B) = 58; U = 1560.5; P = 0.358; one-tailed
5. Neuron#4: N(A) = 24 and N(B) = 89; U = 1052.0; P = 0.574; one-tailed
6. Neuron#5: N(A) = 30 and N(B) = 66; U = 856.5; P = 0.056; one-tailed
7. Neuron#6: N(A) = 28 and N(B) = 45; U = 714.0; P = 0.113; one-tailed
8. Neuron#7: N(A) = 60 and N(B) = 63; U = 1960.5; P = 0.315; one-tailed
9. Neuron#9: N(A) = 85 and N(B) = 75; U = 3106.5; P = 0.346; one-tailed
10. Neuron#32: N(A) = 16 and N(B) = 46; U = 334.0; P = 0.208; one-tailed
11. Neuron#22: N(A) = 20 and N(B) = 9; U = 88.5; P = 0.475; one-tailed
12. Neuron#35: N(A) = 18 and N(B) = 45; U = 416.5; P = 0.587; one-tailed
13. Neuron#10: N(A) = 66 and N(B) = 105; U = 3767.5; P = 0.156; one-tailed
14. Neuron#11: N(A) = 60 and N(B) = 96; U = 3153.0; P = 0.14; one-tailed
15. Neuron#12: N(A) = 74 and N(B) = 72; U = 2496.0; P = 0.212; one-tailed
16. Neuron#13: N(A) = 66 and N(B) = 50; U = 1430.0; P = 0.075; one-tailed
17. Neuron#36: N(A) = 87 and N(B) = 69; U = 3139.0; P = 0.255; one-tailed
18. Neuron#14: N(A) = 94 and N(B) = 75; U = 3567.5; P = 0.432; one-tailed
19. Neuron#15: N(A) = 100 and N(B) = 54; U = 2330.5; P = 0.053; one-tailed
20. Neuron#16: N(A) = 57 and N(B) = 81; U = 2297.5; P = 0.522; one-tailed
21. Neuron#10: N(A) = 15 and N(B) = 48; U = 323.0; P = 0.154; one-tailed
22. Neuron#41: N(A) = 48 and N(B) = 33; U = 696.5; P = 0.145; one-tailed
23. Neuron#42: N(A) = 35 and N(B) = 23; U = 384.5; P = 0.369; one-tailed
24. Neuron#10: N(A) = 15 and N(B) = 124; U = 718.0; P = 0.06; one-tailed
25. Neuron#25: N(A) = 20 and N(B) = 56; U = 652.0; P = 0.117; one-tailed
26. Neuron#46: N(A) = 22 and N(B) = 16; U = 186.0; P = 0.346; one-tailed
27. Neuron#26: N(A) = 77 and N(B) = 76; U = 2675.0; P = 0.145; one-tailed
28. Neuron#47: N(A) = 93 and N(B) = 69; U = 3113.0; P = 0.331; one-tailed
29. Neuron#48: N(A) = 52 and N(B) = 56; U = 1591.0; P = 0.101; one-tailed

C. Mann-Whitney U test results (N=32): Comparing the distribution of evoked spikes preceded by an LFP-SPW coinciding with a spike (group A) with the distribution of evoked spikes preceded by an LFP-SPW without a coinciding spike (group B) + effect size of neurons with alpha < 0.05

No Effect (N=17):

1. Neuron#1: N(A) = 48 and N(B) = 111; U = 2446.5; P = 0.083; one-tailed
2. Neuron#30: N(A) = 46 and N(B) = 88; U = 1844.0; P = 0.188; one-tailed
3. **Neuron#4: N(A) = 21 and N(B) = 92; U = 844.0; P = 0.074; one-tailed**
4. Neuron#5: N(A) = 20 and N(B) = 76; U = 636.5; P = 0.057; one-tailed
5. Neuron#6: N(A) = 27 and N(B) = 46; U = 658.5; P = 0.711; one-tailed
6. Neuron#7: N(A) = 52 and N(B) = 71; U = 1923.5; P = 0.296; one-tailed
7. Neuron#8: N(A) = 36 and N(B) = 42; U = 786.0; P = 0.345; one-tailed
8. Neuron#9: N(A) = 35 and N(B) = 125; U = 2333.0; P = 0.195; one-tailed
9. Neuron#35: N(A) = 17 and N(B) = 46; U = 333.0; P = 0.143; one-tailed
10. Neuron#10: N(A) = 100 and N(B) = 71; U = 3472.5; P = 0.602; one-tailed
11. Neuron#11: N(A) = 95 and N(B) = 61; U = 2485.5; P = 0.052; one-tailed
12. Neuron#36: N(A) = 92 and N(B) = 64; U = 2728.5; P = 0.149; one-tailed

13. Neuron#16: N(A) = 73 and N(B) = 65; U = 2111.5; P = 0.111; one-tailed
14. Neuron#41: N(A) = 28 and N(B) = 53; U = 678.5; P = 0.235; one-tailed
15. Neuron#42: N(A) = 26 and N(B) = 32; U = 320.5; P = 0.057; one-tailed
16. Neuron#44: N(A) = 52 and N(B) = 49; U = 1381.5; P = 0.224; one-tailed
17. Neuron#48: N(A) = 26 and N(B) = 82; U = 985.0; P = 0.186; one-tailed

Excited (N=15):

1. Neuron#3: N(A) = 88 and N(B) = 22; U = 618.0; P = 0.001; one-tailed; r = 0.390
2. Neuron#20: N(A) = 121 and N(B) = 56; U = 2469.0; P = 0.0; one-tailed; r = 0.0325
3. Neuron#34: N(A) = 58 and N(B) = 131; U = 3218.0; P = 0.014; one-tailed; r = 0.0149
4. Neuron#12: N(A) = 58 and N(B) = 88; U = 2092.5; P = 0.012; one-tailed; r = 0.0042
5. Neuron#13: N(A) = 64 and N(B) = 52; U = 1331.0; P = 0.015; one-tailed; r = 0.1500
6. Neuron#14: N(A) = 67 and N(B) = 102; U = 2602.5; P = 0.0; one-tailed; r = -0.1367
7. Neuron#15: N(A) = 124 and N(B) = 30; U = 1421.5; P = 0.01; one-tailed; r = 0.0412
8. Neuron#43: N(A) = 37 and N(B) = 86; U = 1012.0; P = 0.0; one-tailed; r = 0.025
9. Neuron#10: N(A) = 64 and N(B) = 75; U = 1978.0; P = 0.027; one-tailed; r = 0.080
10. Neuron#25: N(A) = 47 and N(B) = 29; U = 516.0; P = 0.026; one-tailed; r = 0.152
11. Neuron#17: N(A) = 22 and N(B) = 87; U = 669.5; P = 0.007; one-tailed; r = 0.050
12. Neuron#18: N(A) = 63 and N(B) = 59; U = 1277.0; P = 0.001; one-tailed; r = 0.415
13. Neuron#26: N(A) = 65 and N(B) = 88; U = 2142.0; P = 0.001; one-tailed; r = 0.280
14. Neuron#47: N(A) = 80 and N(B) = 82; U = 2664.0; P = 0.003; one-tailed; r = 0.122
15. Neuron#19: N(A) = 25 and N(B) = 61; U = 647.5; P = 0.015; one-tailed; r = 0.025
